# Stiffening of the Extrapulmonary Arteries From Rats in Chronic Hypoxic Pulmonary Hypertension

**DOI:** 10.6028/jres.113.018

**Published:** 2008-08-01

**Authors:** E. S Drexler, J. E Bischoff, A. J Slifka, C. N McCowan, T. P Quinn, R Shandas, D. D Ivy, K. R Stenmark

**Affiliations:** National Institute of Standards and Technology, Boulder, CO 80305; Zimmer, Inc., Warsaw, IN 46581-0708; National Institute of Standards and Technology, Boulder, CO 80305; University of Colorado, Boulder, CO 80309; The Children’s Hospital, Denver, CO 80218; University of Colorado Health Sciences Center, Denver, CO 80262

**Keywords:** hypoxia, pulmonary hypertension, rat model, stress, stretch, stiffening

## Abstract

Changes in the compliance properties of large blood vessels are critical determinants of ventricular afterload and ultimately dysfunction. Little is known of the mechanical properties of large vessels exhibiting pulmonary hypertension, particularly the trunk and right main artery. We initiated a study to investigate the influence of chronic hypoxic pulmonary hypertension on the mechanical properties of the extrapulmonary arteries of rats. One group of animals was housed at the equivalent of 5000 m elevation for three weeks and the other held at ambient conditions of ~1600 m. The two groups were matched in age and gender. The animals exposed to hypobaric hypoxia exhibited signs of pulmonary hypertension, as evidenced by an increase in the RV/(LV+S) heart weight ratio. The extrapulmonary arteries of the hypoxic animals were also thicker than those of the control population. Histological examination revealed increased thickness of the media and additional deposits of collagen in the adventitia. The mechanical properties of the trunk, and the right and left main pulmonary arteries were assessed; at a representative pressure (7 kPa), the two populations exhibited different quantities of stretch for each section. At higher pressures we noted less deformation among the arteries from hypoxic animals as compared with controls. A four-parameter constitutive model was employed to fit and analyze the data. We conclude that chronic hypoxic pulmonary hypertension is associated with a stiffening of all the extrapulmonary arteries.

## 1. Introduction

Traditionally, pulmonary arterial hypertension is thought of as a disease of the peripheral lung vasculature, wherein excessive vasoconstriction and/or structural remodeling of the small pulmonary blood vessels are the major contributors to increased pulmonary vascular resistance and right ventricular overload. However, it is increasingly appreciated that changes in the properties affecting the capacity of large blood vessels may also be critical determinants and/or contributors to the increases in afterload that contribute to right heart dysfunction and ultimately right heart failure in the setting of pulmonary hypertension [1–4]. Indeed, the importance of the decreased vascular wall compliance (stiffening) of large vessels has been highlighted by studies demonstrating that impaired pulsatile arterial function is an independent predictor of risk for cardiovascular events for many diseases including pulmonary arterial hypertension [5–7]. In fact, assessment of the dynamic (capacitance or compliance) components of the arterial system has been suggested to provide important prognostic and therapeutic information beyond that provided by traditional measurements of blood pressure or vascular resistance [7–9]. Under normal conditions, the elastic conduit vessels constitute a hydraulic buffer that acts to convert the intermittent cardiac output into steady flow that not only reduces cardiac workload during systole and conserves energy expenditure for the heart, but also alleviates pulsatile stress for the perfused organs. Stiffening of the conduit arteries increases the pulsatile component, exerting detrimental hemodynamic load on the heart, limiting coronary perfusion, impairing ventricular performance and eventually exacerbating development of heart failure [7–10]. However, the relationship of this remodeling to increases in pulmonary vascular resistance is controversial [10,11]. Thus, better insight into the question as to whether or not pulmonary hypertension causes the stiffening of the proximal vessels and ultimately as to what causes this is important for a better understanding of pulmonary hypertension and associated right ventricular dysfunction.

Numerous animal models have been utilized to study pulmonary hypertension. One commonly utilized model system is exposure of animals to chronic hypoxic conditions, which causes structural remodeling in both large and small vessels [10]. Importantly, it has been demonstrated that chronic hypoxic pulmonary hypertension in mice is associated with increases in impedance and in the stiffness of the left main pulmonary artery [12–14]. Rats also develop pulmonary hypertension in response to chronic hypoxia, and the magnitude of the pressure changes, vascular remodeling, and right heart hypertrophy appear to be greater than those in mice [10]. However, the effects of chronic hypoxia on vascular stiffening in all three proximal conduit vessels of rats have, to our knowledge, not been previously investigated.

The objective of this study, therefore, is to compare the passive mechanical properties of the proximal pulmonary arteries in chronically hypoxic rats to those of control animals housed at ambient conditions to determine the extent to which hypoxic pulmonary hypertension causes stiffening of the conduit vessels. Our use of a bubble inflation technique, which we have previously reported as useful in the study of passive mechanical properties [15], is one that is not limited by the length or curvature of the material. Therefore, this study will determine whether all extrapulmonary arterial vessels, including the trunk and the right and left main arteries, are equally susceptible to the development of stiffening in response to chronic hypoxia.

## 2. Materials and Methods

Male rats from a breeding colony of the Long-Evans strain were housed and euthanized at the University of Colorado Health Sciences Center according to the Animal Care and Use Committee approved protocol ASP #44404004(07)1E. This strain of rats was used because they were readily available. (All animals discussed here were +/+ for the endothelin B receptor [16].) The control animals (n = 7) were housed at ambient pressure in Denver, Colorado (elevation ~1600 m) throughout their lifetimes and were 12 weeks, 2 days old (mean age ± 2 days) at the time of death. The experimental rats (n = 6) were housed for 3 weeks in a hypobaric chamber at the equivalent of 5000 m in elevation and were 12 weeks, 3 days old (mean age ± 5 days) at the time of death. Following euthanasia, the heart and lungs were dissected from the thorax. The trunk and main pulmonary arteries were isolated and placed in a nutrient-balanced medium on ice until the time of study (always within 24 h of excision). The heart was dissected to determine the ratio of weights of the right ventricle to the left ventricle plus septum [RV/(LV+S)].

The arteries were first visually inspected for anomalies (holes, tears, extraneous connective tissue, etc.); then each artery section was removed and prepared further to provide three test specimens per animal—one from each, the trunk, right main artery, and left main artery. Each section was cut parallel to its axis and opened to form a rectangular piece of material. A reference line was marked on the material with India ink, indicating the direction of blood flow through the artery. From the rectangle, a circular disk (~3 mm in diameter) was cut for placement into the test fixture. The test specimen was then placed intima side up on an O-ring and loaded into the fixture with the flow (longitudinal) direction of the specimen aligned so that it corresponded to the view from our 0° camera. Care was taken to avoid prestressing the specimen during this procedure. For the duration of the test, the test fixture was placed in a reservoir of phosphate-buffered saline (PBS) solution (without Ca or Mg), and held at 37 °C. The initial pressure for the test was determined by inflating the tissue to 3.45 kPa, deflating it until the tissue retracted below the horizontal surface of the fixture, then slowly increasing the pressure to what we refer to as the instability pressure point. This is defined as the pressure at which the bubble continued to expand with no additional increase in pressure. Since the material inflated without additional pressurization, it appears that the tissue was achieving its natural degree of curvature, which must correspond to the state of zero applied external stress. This instability point was unique to the individual specimen, controlled by the degree of curvature in the tissue and/or the degree of slack during placement. We consider that for all the tests, the tissue was in its naturally relaxed state because the test section was cut open and the test was started at this instability point.

Specifics of the experimental setup were previously described [17]. The cylindrical tank was designed with twelve equally spaced optical glass windows at 30° intervals, through which the specimen may be viewed. A set of six images were taken of the inflated material by three cameras, 60° apart, and with a rotating stage. All elements of the pressurization system had low compliance relative to the test material, and the range of pressure was adequate for testing of the compliant arteries with minimal pressure hysteresis.

The test was automated through the use of commercially available computer software to incrementally increase pressure at prescribed intervals, collect images from the three cameras, and control the position of the rotating stage holding the fluid reservoir and test fixture. The cameras, positioned to acquire images of the bubble profile, collected images every 1.38 kPa (10 mmHg) in the neutral position and at 30° rotation. To ensure complete characterization of the nonlinear behavior, a maximum inflation pressure of 17.9 kPa (135 mmHg) was chosen for the arteries from the control animals and 27.6 kPa (207 mmHg) for the hypoxic animals. Upon completion of the test, the specimen was deflated and removed from the test fixture for measurement of thickness. Due to the highly elastic nature of this material, the thickness should be unaffected by the effects of pressurization. The thickness could not be measured prior to the test because of the likelihood that the pressure-deformation behavior would be adversely affected by dehydration of the tissue.

The thickness of each test specimen was measured after the test with a laser micrometer. As many measurements (typically 10 to 12) as could be recorded within 30 s at different locations on the artery were made for each specimen and averaged to determine the material thickness. Care was taken to make sure that the specimen remained moist but free of fluid beading on the surface, as either condition could affect the measured thickness.

Representative animals from each population were sacrificed specifically for histology. The trunk and lobar arteries were fixed in formalin and mounted in a pre-embedding gel, then paraffin. Following thin sectioning, they were stained with pentachrome. The sections were evaluated by digital image analysis for the following variables: (1) thickness of the individual elastic lamella, (2) medial thickness, (3) perimeter of inside (intima) and outside diameter (external elastic lamella), and (4) area fraction of elastin (including lamella and elastin within matrix). Evaluations were conducted at locations 12, 2, 4, 6, 8, and 10 o’clock on the cross sections of the whole arteries. Data are presented as a function of the percent medial thickness, normalized by the outside diameter of the artery to eliminate biases that may be introduced by an artery that is proportionally large or small.

## 3. Analysis

**Fig. 1:**
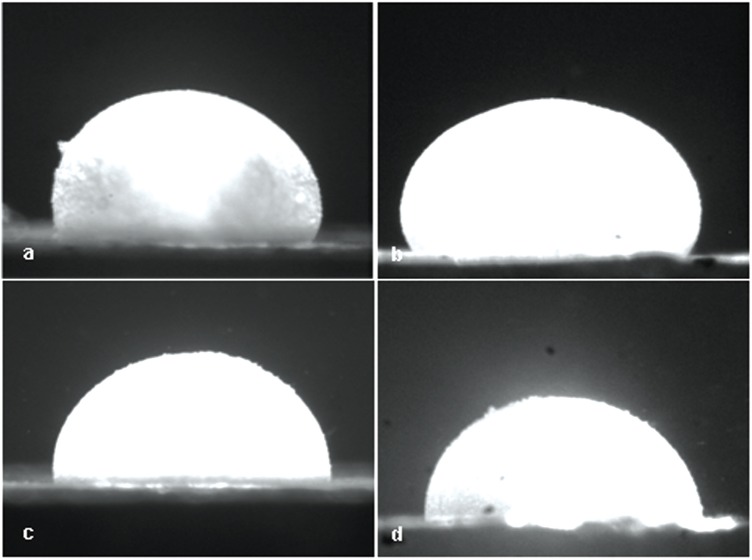
Images showing a fully inflated artery specimen from a control trunk, viewing displacements (a) in the circumferential orientation, and (b) in the direction of blood flow, as compared with a hypoxic trunk inflated to the same pressure in the (c) circumferential orientation and (d) direction of blood flow. Images show anisotropy in the deformation.

Raw data were in the form of six side-view images for every pressure increment, similar to those shown in Fig. 1. From those images the length of the bubble profile was measured and the average stretch was calculated. The orientation that displayed the smallest bubble diameter (minor axis) was assumed to correlate with the stiffer in-plane material orientation; the orthogonal orientation was, therefore, taken to be the most compliant in-plane material orientation. The most common orientation for the minor axis corresponded to the longitudinal/flow direction for the main arteries and the circumferential direction in the trunks.

The most straightforward comparison that can be made between these two populations is found by measuring the deformation with respect to pressure. In a clinical setting, pressure is measured and arteries can be imaged to obtain distensibility [18]. For the purposes of comparison, an average stretch (hereafter referred to as stretch) was calculated by measuring the entire length of the bubble profile in a particular orientation, divided by the original length of the bubble profile (*λ* = *l*/*l*_o_). This comparison is valid since the shape of the bubble is similar in both populations, although absolute sizes differ. The stretch of the section of the artery at a representative elevated pressure, indicates whether the distensibility is impaired as the result of chronic hypoxia.

Also calculated for each section of the two populations was the initial modulus. We defined this to be the slope of the stress (σ)-strain (ε) curve for 0 ≤ ε ≤ 0.2. The σ and ε were calculated as described in [15].

In order to relate the mechanical data to fundamental material parameters, an inverse finite element analysis was performed for each set of experimental results. A simple model of the structure created when the tissue is inflated was developed: a zero displacement boundary condition was used around the edge of the aperture with a pressure boundary condition on one side of the tissue. Details about this process, including the governing constitutive law, the computational model of membrane inflation, and the regression process for estimating the material parameters, are fully described in a companion paper [19]. Here, key aspects of this analysis will be summarized.

Given the general biomechanical characteristics of the arterial sections (nonlinear, anisotropic material behavior), as well as prior success with similar data sets [15,20], an adaptation of the orthotropic, nonlinear elastic model of Bischoff et al. [21,22] was used to describe the mechanical behavior of the arterial tissue. Assuming incompressibility, this model utilizes four independent material parameters (*E*_0_, *A*, *B*, *P*). The model is expressed mathematically by a unit cell made up of crimped fibers connecting the corners of the unit cell, representing the stiffening mechanism in that orientation (collagen, for example). If the unit cell is extended in either in-plane orientation, the fibers will extend until they have reached their maximum length. The parameter *E*_0_ is related to the local fiber density in the tissue, and the parameters *A* and *B* are related to the degree of fiber alignment for each in-plane orientation (longitudinal and circumferential). The parameter *P* is correlated with the locking stretch of the tissue, which corresponds to the knee in the pressure-stretch, pressure-strain, or stress-strain curves.

Parameters of the constitutive model were determined by comparison of results from finite element simulations of the bubble inflation tests with the experimental bubble pressure versus strain data using ABAQUS 6.7-1, a commercially available general purpose finite element package.^1^ These parameters were determined by comparison of the average stretch estimated from the experimental images in the respective directions and the average stretch calculated from the finite element simulations for the corresponding directions. In particular, the orientations of the material axes, bubble strains as viewed along the longitudinal and circumferential axes, and pressure were used as input for homogeneous parameter regression. Unlike previous work [20] in which parameters were varied through trial and error until a reasonable fit was obtained, an automated inverse finite element process was utilized here. Details of this process can be found in the companion paper [19], in which both global and local optimization approaches are addressed. Here, an initial set of material parameters was determined using either trial and error or a global optimization algorithm. Then, a local optimization algorithm (quadratic programming) was employed to determine the set of parameters which best captured the data. The objective function for the optimization was the sum of the *R*^2^ measure between the experimental data and the model predictions in each in-plane direction. A value of *R*^2^ = 2 therefore represents perfect agreement between model and data; the mean value across all data sets considered here was *R*^2^ = 1.947, ranging between 1.712 and 1.997.

ANOVA was performed using a Student’s *T* test on various parameters to identify differences between the populations and between any two of the sections. A paired *T* test was employed when comparing different sections from the hypoxia-treated population, as the data for each section came from the same animals. Significance was defined as *p* < 0.05.

## 4. Results

**Fig. 2:**
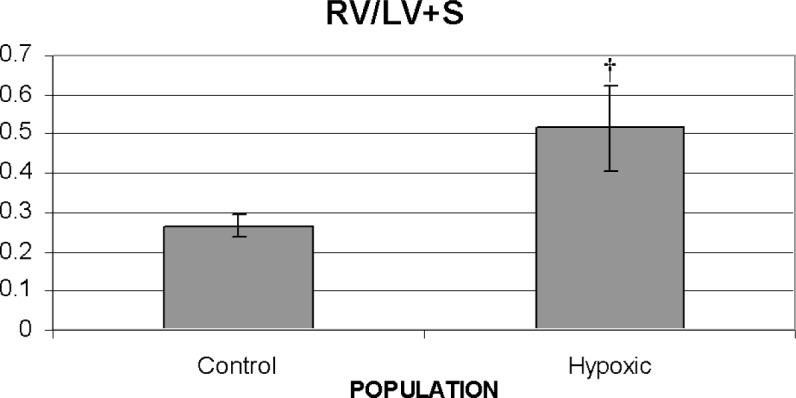
Comparison of mean values for RV/LV+S for the control and hypoxic populations. Standard deviations are shown with error bars. ^†^*p* < 0.005.

**Fig. 3:**
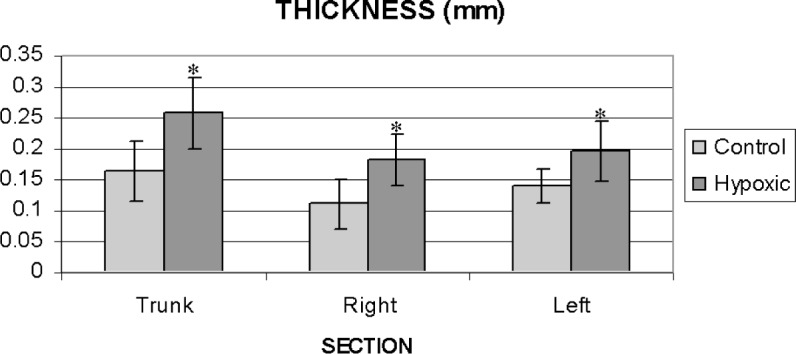
Comparison of mean values for wall thickness of the trunk, right and left main arteries for the control and hypoxic populations. Standard deviations are shown with error bars. *p < 0.05.

Chronic hypoxic exposure (3 wks) led to the development of pulmonary hypertension as evidenced by the increase in the RV/LV+S ratio (Fig. 2). In addition, we found that chronic hypoxic exposure led to a thickening of each arterial segment examined, i.e., the trunk, right and left main pulmonary arteries (Fig. 3). No significant differences in thickness among the three arterial segments were noted under either control or hypoxic conditions (Fig. 3).

**Fig. 4:**
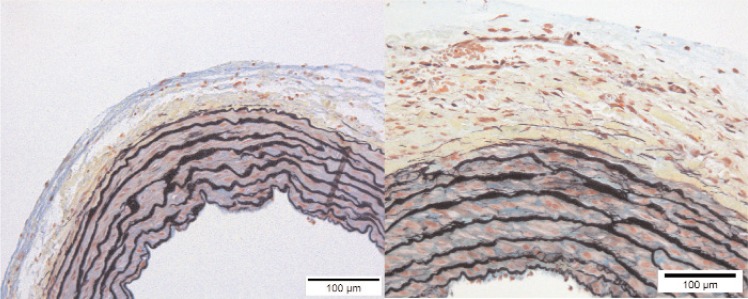
Images showing histology of a control trunk (left) and a hypoxic trunk (right). The thicknesses of the media at this location are about 120 μm and 160 μm, respectively. This hypoxic trunk shows denser collagen and pseudo lamellae in the layer of the adventitia adjacent to the media (pentachrome stain).

**Fig. 5:**
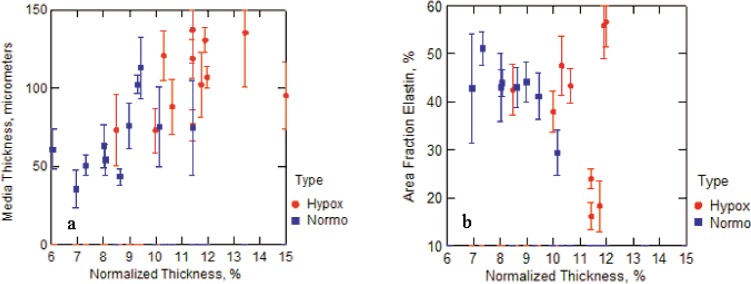
Media thickness versus normalized (by the diameter) thickness (a) and areal fraction of elastin versus normalized diameter (b) for each section of control and hypoxic extrapulmonary arteries. Evaluations were conducted at locations 12, 2, 4, 6, 8, and 10 o’clock on the cross sections of the whole arteries. Error bars represent one standard deviation.

Histological analysis provided an additional means of determining how and where the arterial walls thickened, as shown in Fig. 4. Measurements from the cross sections show the thickness of the media ranged between 7 % and 16 % of the diameter for both trunk and main arteries, as shown in Fig. 5a. The values for the control arteries were 7 % to 9 %, which is near the upper range of the 3 % to 7 % reported by Kay and Heath [23] for normal rats at sea level. Comparatively, the thickness values of 10 % to 16 % measured for the hypoxic samples indicate thickening of the media, as shown in Fig. 5a. The increased thickness of the medial layer is mostly due to thicker muscular layers. The areal-fraction of elastic fiber, associated with the elastic lamellae, does not appear to have a strong correlation with hypertension, as shown in Fig. 5b. The medial thickness and areal-fraction of elastin are plotted against the percent of the medial thickness normalized by the diameter in order to account for differing sizes of rats and their arteries.

**Fig. 6:**
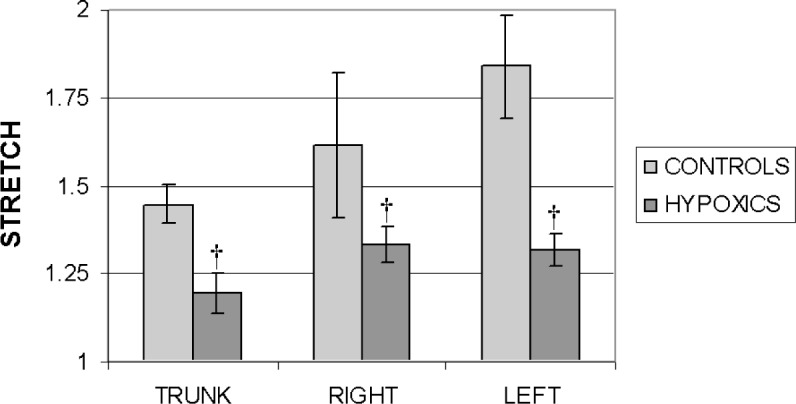
Comparison of mean values for stretch (*λ* = *l*/*l*_0_) at inflation pressure = 7 kPa (53 mm Hg) of the trunk, right and left main arteries for the control and hypoxic populations. Standard deviations are shown with error bars. ^†^*p* < 0.005.

**Fig. 7:**
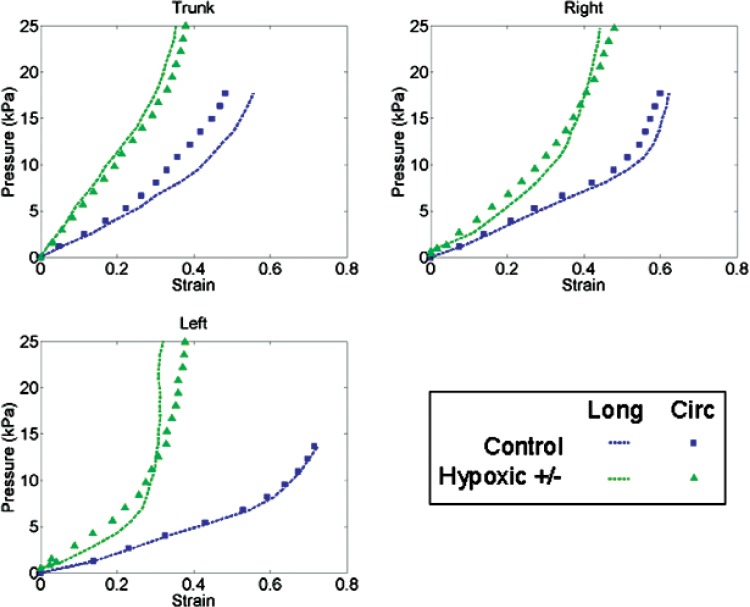
Representative sets of pressure versus strain data from each population and each arterial region, in both the longitudinal (“Long”) and circumferential (“Circ”) directions. Note that the initial stiffness of the trunk specimens (related to the linear portion of the pressure versus strain curve) is generally greater than the stiffness from the other arterial sections (right and left) of the same population.

Figure 6 shows the comparison of the mean value for the stretch for each population and arterial section from the control and hypoxic populations at a representative pressure. A pressure of 7 kPa (53 mmHg) was chosen. This pressure was supported by all of the samples tested. It is clear from the bar graphs that, at the same pressure, the arteries from the chronically hypoxic rats, from every section, display less stretch than do those from the control animals. An alternate way of visualizing the stiffness behavior is with plots of pressure versus strain. Figure 7 shows representative pressure versus strain data from each population and arterial segment in the longitudinal (long) and circumferential (circ) orientations. The increase in stiffness of all arterial segments from the hypoxic animals is readily apparent; these plots of pressure versus strain depict distinct differences in behavior between arteries tested from control and hypoxic animals. Although pressure does not take into account the increased thickness of the arterial wall observed in Fig. 3 due to chronic hypoxia, whether data is plotted as pressure versus strain, stress (as calculated in [15]) versus strain, or simply as the pressure normalized by the thickness versus strain, for all methods the arteries from chronically hypoxic rats exhibit less strain than their control counterparts.

**Fig. 8:**
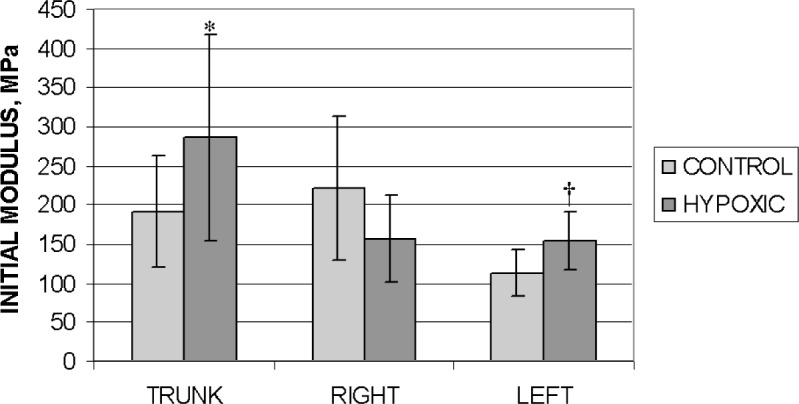
Comparison of mean values for initial modulus of the trunk, right and left main arteries for the control and hypoxic populations. The trunk and left main arteries from the hypoxic population are statistically different from those of the control population. Standard deviations are shown with error bars. *p < 0.05, ^†^*p* < 0.005.

We define the initial modulus as the slope of the σ-ε curve at strains from 0 to 0.2. A maximum value for strain of 0.2 was chosen because it was the limit of the linear region for the majority of the tests. To calculate the mean value for the initial modulus, we used the slopes from both orientations of all the tests from a given section within a population. In Fig. 8, we show that this initial modulus is significantly different when comparing data from control and hypoxic animals, but only for the trunk and the left main arteries (*p* < 0.05 and *p* < 0.005, respectively). Significance was not demonstrated for the right main artery.

**Fig. 9:**
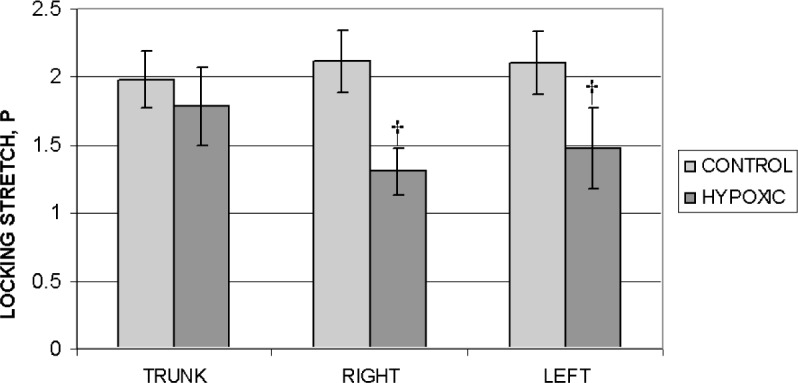
Comparison of mean values for the locking stretch *P* of the trunk, right and left main arteries for the control and hypoxic populations. The right and left main arteries from the hypoxic population are statistically different from that of the control population. Standard deviations are shown with error bars. ^†^*p* < 0.005.

The constitutive model used provides parameters with which to statistically analyze the differences in mechanical properties between arteries from control versus hypoxic animals. The locking stretch *P* can qualitatively be described as corresponding to the onset of strain stiffening, or where the knee occurs in the stress-strain curve. The variable *P* is statistically compared in Fig. 9 for the different sections of the extrapulmonary artery from control and chronically hypoxic rats. The differences are statistically significant for the right and left main arteries (*p* < 0.005), but are not the trunks.

Anisotropy *α*, defined as the maximum deviation in the expansion of the inflated tissue bubble in any paired orientations—ellipticity of the tissue bubble—was also evaluated for this suite of tests. The statistical comparison of anisotropy was conducted by measuring the difference in diameters of the bubble in the major and minor axes (*α* = *a* − *b*). Comparisons were made among sections and between populations and there appeared to be no statistical significance (not shown), although individually bubbles exhibited anisotropy (Fig. 1). The values for *α* ranged between 0.06 mm and 0.55 mm, with the mean for all the tests of 0.24 mm.

The instability pressures were also compared to assess possible effects of pulmonary hypertension with its attendant remodeling of the arterial walls. We found no significant differences in the instability pressure for any section or population (not shown).

## 5. Discussion

We tested the hypothesis that chronic exposure of rats to hypoxia would cause a stiffening of the extrapulmonary arteries. Using our previously established bubble inflation technique [17], we found that hypoxic pulmonary hypertension was associated with a variety of changes in tissue behavior in the trunk, right and left main pulmonary arteries that all supported a stiffening of these vessels. Changes in distensibility, stress-strain relations, locking stretch, and initial modulus were observed in tissue segments from chronically hypoxic extrapulmonary arterial tissue. The collective changes in mechanical properties of hypoxic vessels are consistent with stiffening changes observed in large vessels of humans with pulmonary hypertension and in chronically hypoxic mice [4,12–14]. Thus, it is increasingly clear that pulmonary hypertension is associated with marked functional changes in the extrapulmonary vessels. These changes can contribute to right ventricular dysfunction, which is known to be the major determinant of morbidity [3].

The measure of stretch at a defined pressure proved to be the most sensitive to differences in mechanical properties between the control versus hypoxic vessels. The equivalent effect is a reduction in the distensibility observed in pulmonary arteries due to chronic hypoxia [14,24]. When viewed as stress-strain, in response to chronic hypoxia, all arterial segments tested displayed much less strain before stiffening occurred; that is, the knee in the stress-strain curve occurs at lower strains in the vessels from the chronically hypoxic animals. These changes are consistent with the previously reported changes in the pressure-diameter curves for the large vessels observed in the setting of chronic pulmonary hypertension in the human [4].

As has been previously demonstrated by others, we found evidence of remodeling in the extrapulmonary arteries of the chronically hypoxic rats [10]. All the extrapulmonary arteries studied from the chronically hypoxic animals had a thicker media and changes in the adventitia (which included thickening and, based on the pentachrome staining, increases in collagen). Previous studies in the chronically hypoxic rat as well as in the mouse have demonstrated thickening of the left main artery and a marked increase in collagen deposition [13]. At a constant pressure, increases in thickness and in collagen content might be expected to contribute to a stiffer response. Indeed this has been demonstrated in the stiffening responses seen in systemic vessels in a number of conditions. Furthermore, the Chesler group has demonstrated that, at least in the pulmonary arteries of a hypoxic mouse, the accumulation of collagen, particularly in the adventitia, is a strong predictor of the stiffening response observed in the hypoxic pulmonary hypertensive vessel [13]. The data presented in Fig. 5 are a small sampling. We recognize that these results are based on a small population of rats and may not be representative of a greater population, nor is individual variability necessarily sampled. More studies are necessary to determine all the factors contributing to the marked stiffening observed in the extrapulmonary vessels in response to chronic hypoxic exposure.

The initial modulus of the trunk and the left main extrapulmonary artery is significantly different in the chronically hypoxic animals as compared with those from the control animals. Hypoxia induces an increase in the initial modulus in the trunk and the left main arteries, but not in the mean value of the right main pulmonary artery. Studies in mice also showed differences in the initial modulus of the left main artery between control and hypoxia-exposed mice that were dynamically loaded [13]. The initial-modulus behavior of the right main artery is confusing, especially as we see no differences histologically or in the thickness to account for a smaller initial modulus in the chronically hypoxic population. With limited published data on the right main pulmonary artery, we are unable to determine if this unexpected behavior is observed by others.

Remodeling is the expected result of pulmonary hypertension and the literature would indicate that changes in fibers of the extracellular matrix contribute to the remodeling [10,25–30]. These changes in fiber alignment and density would directly affect the strain-stiffening behavior. The variable *P*, derived from the four-parameter model, provides an excellent means of comparing the control and hypoxic datasets when strain-stiffening is a prominent feature. Such is the case with the right main arteries (*p* < 0.00005) and, to a lesser degree, the left main arteries (*p* < 0.005). Most of the main arteries from the rats exposed to chronic hypoxia exhibited increases in stress with no obvious increase in strain by the end of the test. Nearly all of the tests from the control animals and the hypoxic trunks still exhibited increases in strain with increased stress at the end of the test, although the degree of strain was less in the hypoxic trunks. This indicates that, although less distensible than those from the control animals, the trunks from the chronically hypoxic rats maintain a greater degree of elasticity than do the main arteries.

The most common direction for the minor axis of deformation for the main arteries as compared with the trunks is 90° apart, consistent for both populations. This would suggest that the sections perform different functions. Since the main arteries can accommodate larger displacements in the circumferential direction, it follows that the main arteries play a larger role in pulsatile dampening. Differences in the strain-stiffening behavior between the trunks and main arteries may also be attributable to these functional differences. Compensating for the higher arterial pressures of pulmonary hypertension may affect the more compliant, pulsatile-dampening main arteries more than the trunks, consequently resulting in greater degrees of strain-stiffening.

The control group is somewhat unique, as six of the nine animals tested were litter mates, and we attribute the smaller standard deviations to this consistency in genetic makeup.

There are certain limitations to the current study. Although the control animals were housed at ambient conditions in Denver, CO, the RV/LV+S data fell within the range typical of normoxic animals (see Table 1 in [31]), and it appears that the animals did not develop pulmonary hypertension from being housed at ~1600 m. The significantly (*P* ≤ 0.005) increased values for RV/LV+S within the treated population, as compared with our control animals, demonstrate that we are comparing tissue from chronically normotensive and hypertensive animals. It is also recognized that the bubble inflation technique does not resemble physiological conditions. The technique more commonly seen in the literature is that in which a segment of artery is cannulated and inflated, with changes in diameter recorded with respect to pressure. That technique more closely resembles physiological conditions. However, it requires a straight segment of artery and it must be sufficiently long to cannulate. Furthermore, resolving displacements from the direction of blood flow is not trivial. The bubble inflation technique, however, provides data from different orientations (specifically the direction of blood flow and the circumferential orientations, for this study), and is more adaptable, allowing us to obtain properties from all three sections of the extrapulmonary system: the trunk, right, and left main arteries. With the proper analysis of the images, accurate calculations of the deformations due to pressure are possible, which can be related to fundamental material properties using finite element analysis, both allowing comparisons and detecting differences in the mechanical properties of the pulmonary arteries of control and hypoxic rats.

## Footnotes

1 Certain commercial equipment, instruments, or materials are identified in this paper to foster understanding. Such identification does not imply recommendation or endorsement by the National Institute of Standards and Technology, nor does it imply that the materials or equipment identified are necessarily the best available for the purpose.
